# Assessment of microplastic pollution: occurrence and characterisation in Vesijärvi lake and Pikku Vesijärvi pond, Finland

**DOI:** 10.1007/s10661-019-7843-z

**Published:** 2019-10-18

**Authors:** Costanza Scopetani, David Chelazzi, Alessandra Cincinelli, Maranda Esterhuizen-Londt

**Affiliations:** 1 Faculty of Biological and Environmental Sciences, Ecosystems and Environment Research Programme, University of Helsinki, Niemenkatu 73, FI-15140 Lahti, Finland; 2 Department of Chemistry “Ugo Schiff”, University of Florence and Consorzio Interuniversitario per lo Sviluppo dei Sistemi a Grande Interfase (CSGI), Sesto Fiorentino, 50019 Florence, Italy; 3 Department of Chemistry “Ugo Schiff”, University of Florence, Sesto Fiorentino, 50019 Florence, Italy; 4 Helsinki Institute of Sustainability (HELSUS), Fabianinkatu 33, 00014 Helsinki, Finland; 5 Environmental Safety Group, Joint Laboratory of Applied Ecotoxicology Campus E 7.1, Korean Institute of Science & Technology (KIST Europe), 66123 Saarbrücken, Germany

**Keywords:** Environmental monitoring, Microplastics, Vesijärvi lake, Freshwater environments, Microplastic quantification

## Abstract

In the last few years, several studies have investigated microplastics (MPs) in marine ecosystems, but data monitoring and assessing the occurrence in freshwater environments are still scarce. The present study aims to investigate the occurrence, distribution, and chemical composition of MP pollution in Vesijärvi lake and Pikku Vesijärvi pond close to the city of Lahti (Finland) in winter. Sediment, snow, and ice core samples were collected near the shore of these two aquatic systems. MPs were analysed and identified by a non-destructive method using Fourier transform infrared spectroscopy (FTIR) 2D imaging. The mean concentrations of MPs detected in sediment, snow, and ice samples were 395.5 ± 90.7 MPs/kg, 117.1 ± 18.4 MPs/L, and 7.8 ± 1.2 MPs/L, respectively. FTIR results showed the predominant abundance of microplastics, such as polyamides (up to 53.3%), polyethylene and polypropylene (up to 17.1%), and natural fragments such as cellulose (up to 45.8%) and wool (up 18.8%) in the same size range. The potential release of MPs arising from stormwaters and sport and recreational activities was evidenced.

## Introduction

Plastic litter discharged into the environment is a global issue recognised as an emerging threat by the scientific community (Eerkes-Medrano et al. 2015). A remarkable amount of plastic waste discharged in the aquatic systems is in the form of microplastics (MPs), polymeric particles smaller than 5 mm (US EPA 2011), originated from the breakdown of larger plastic objects (secondary MPs) or originally manufactured as pellets for the production of plastic items or as granules to add to personal care products (primary MPs). MPs are found everywhere in marine environments (oceans, shorelines, sediments, surface waters) (Thompson et al. 2009; Hidalgo-Ruz et al. 2012; Van Cauwenberghe et al. 2013; Isobe et al. 2017; Cincinelli et al. 2018; Martellini et al. 2018; Ivleva et al. 2016; Yu et al. 2018) even in the remotest areas of the world like the Arctic ice and Antarctic waters (Bergmann et al. 2017; Cincinelli et al. 2017). MPs have even been detected in marine biota, including seafood species (Van Cauwenberghe and Janssen 2014; Pegado et al. 2018).

Comparing the number of MP studies conducted in the marine environment with the ones regarding freshwater systems, a remarkable discrepancy can be observed. Within MP studies, less than 4% concerns freshwater environment (Lambert and Wagner 2018), and thus, there is a current need to collect data to establish the occurrence and the impact of MPs in these aqueous systems.

Research conducted on freshwater ecosystems shows that MP pollution seems to be ubiquitous, and the concentrations are similar to those found in the marine environment (Lambert and Wagner 2018; Klein et al. 2018). Reported MP concentrations in surface water samples of 20 urban lakes and urban reaches of Hanjiang River and Yangtze River of Wuhan (China) ranged from 1660 ± 639 to 8925 ± 1591 pieces/m^3^ with an increasing concentration closer to the city centre (Wang et al. 2017). Fibres were the most abundant MPs found in the samples, and polypropylene and polyethylene terephthalate were the prevalent polymers.

Another study investigated MP occurrence in Taihu lake (Su et al. 2016) which is the third largest freshwater Chinese lake and is considered one of the most polluted due to several industrial and touristic activities surrounding it. Su et al. (2016) detected MPs in the concentration range of 3.4 to 25.8 pieces/L in surface water and 11.0 to 234.6 pieces/kg dry weight (dw) in sediment samples.

A study conducted in Lake Garda (Italy) (Imhof et al. 2013) reported MPs in lakeshore sediments ranging from 108 to 1108 piece/m^2^ with a remarkable inhomogeneity between north and south lakeside; in the river Danube, the concentration of plastic items exceeded that of larval fish (Lechner et al. 2014).

The high abundance of MPs is particularly alarming because they can be ingested by organisms such as zooplankton, fish larvae, invertebrates, fish, and birds (Teuten et al. 2007; Ugolini et al. 2013; Cole et al. 2015; Mazurais et al. 2015; Lusher et al. 2016; Zhao et al. 2016; Caron et al. 2018; Scopetani et al. 2018). MP ingestion could lead to physical damage (Wright et al. 2013) and cause an illusory sense of satiation that may alter the feeding behaviour (Gregory 2009; Cole 2014). Moreover, ingested MPs can leach and transfer toxic additives, such as plasticisers and other organic chemicals adsorbed from the environment to the biota, posing a potential health hazard (Teuten et al. 2009).

Monitoring campaigns are essential to understand the extent of MP presence in freshwater environments, and the present study aims to investigate the occurrence and distribution of MP pollution in urban Vesijärvi lake and Pikku Vesijärvi pond, close to the city of Lahti (Finland). Lake Vesijärvi has been the subject of a successful restoration project, which since the middle of 1970 diverted wastewater of the treatment plants of the city of Lahti from the lake (Horpilla and Kairesalo 1990); nowadays, the water basin only receives stormwater from the city centre.

Vesijärvi lake and Pikku Vesijärvi pond represent common meeting points for tourists and citizens of Lahti during the wintertime for walking, skating, skiing, and sledging on its frozen surface.

Thus, the aim of the current study was to correlate the MP characterisation to possible local sources of MP pollution by sampling sediment, snow, and ice samples and analysing the MP types and quantities using Fourier transform infrared spectroscopy (FTIR).

Sediment, snow, and ice core samples were collected near the shore of the two lakes. To avoid any contamination of plastic fibres coming from clothes, the working coveralls worn by the samplers were made entirely from red, natural fibres such as cotton and leather. Ice core and snow samples were directly filtered on glass fibres, while MPs in sediment samples were first extracted through density separation. To minimise the potential risk of contamination, the filters were analysed, without any additional treatment, with a non-destructive method consisting of a focal plane array (FPA) FTIR 2D imaging. The FPA detector has been successfully used by Cincinelli et al. (2017) to identify MPs on complex organic samples without the need to separate MPs from the filters. Thanks to their enhanced spatial resolutions (from ca. 1 to 5 μm), FPA detection has been recognised as a robust and suitable method to analyse MPs (Löder et al. 2015; Tagg et al. 2015).

## Materials and methods

### Sampling

Sediment, snow, and ice core samples were collected near the shore of Vesijärvi lake and Pikku Vesijärvi pond, close to the city of Lahti (Finland). Sampling was conducted according to Pierre Gy’s theory of sampling principles (Pitard 1993) to collect composite samples near the shore (± 100 to 200 m) of lake Vesijärvi (coordinates system ETRS-TN35FIN: N 6764247.147 E 426083.837), and near the shore (± 20 to 30 m) of pond Pikku Vesijärvi (coordinates system ETRS-TN35FIN: N 6762313.197 E 426766.529. The samples were collected from the two sites in March 2018. Snow samples were collected from the top layer (first 5 cm of depth) using a metal spoon and stored in glass jars. Ice core samples were collected with a metallic ice drill and then placed in metallic buckets covered with aluminium lids. Sediments (max first 5 cm of depth) were sampled lowering an Ekman sampler into the ice holes previously drilled, and then they were stored in glass jars.

### Sample treatment and FTIR analyses

Snow and ice core samples were melted and then directly filtered on glass microfibres (grade 693, 90 mm diameter, particle retention 1.2 μm, VWR), while MPs in sediment samples were first extracted through a density separation adding a saturated NaCl solution, followed by 10 min of shaking. The sediments were then allowed to settle and the supernatant filtered. To achieve a high MP recovery, the extraction procedure was repeated three times, according to Kovač et al. (2015).

The filters were dried and analysed using a Cary 620–670 FTIR microscope, equipped with an FPA 128 × 128 detector (Agilent Technologies), which allows performing 2D imaging FTIR analysis with the highest spatial resolution currently available to FTIR microscopes. One hundred twenty-eight scans were acquired for each spectrum, in reflectance mode with an open aperture and a spatial resolution of 8 cm^−1^. Each MP analysis consists in a map of 700 μm × 700 μm (128 × 128 pixel) with a spatial resolution of 5.5 μm (i.e. each pixel has a dimension of 5.5 μm × 5.5 μm).

MPs were counted through visual identification and analysed directly on dry glass microfibres, without any further treatment. Fibres and fragments were counted and analysed in five randomly selected squares 2 cm × 2 cm on each filter, total area 20 cm^2^, which comprised about 31.4% of the total filtered area. The results were normalised to the total filter surface area. The final number of MPs was calculated by removing the verified non-plastics.

### Contamination control

To prevent any contamination of synthetic fabrics coming from clothes, during the sampling campaign and laboratory procedures, a working coverall made entirely from red, natural fibres as cotton and leather was worn. Plastic equipment was carefully avoided, and before performing the sampling and the filtration of the samples, all the tools, including the glass jars and the metal buckets, were rinsed with milli-Q water before covering them with aluminium foil.

Procedural blanks were performed to check for any potential contamination coming from sample processing and analysis. Slightly wet glass microfilters were left open to the air in the laboratory and checked for airborne contamination. All the analytical steps were performed under a laminar flow cupboard. Airborne laboratory contamination was minimised, avoiding any potentially invasive pre-treatment of the samples that could also alter the size and the shape of MPs.

## Results and discussion

### Microplastic characterisation

MPs were detected in all three sample matrices, together with items made of cellulose and wool.

**Fig. 1:**
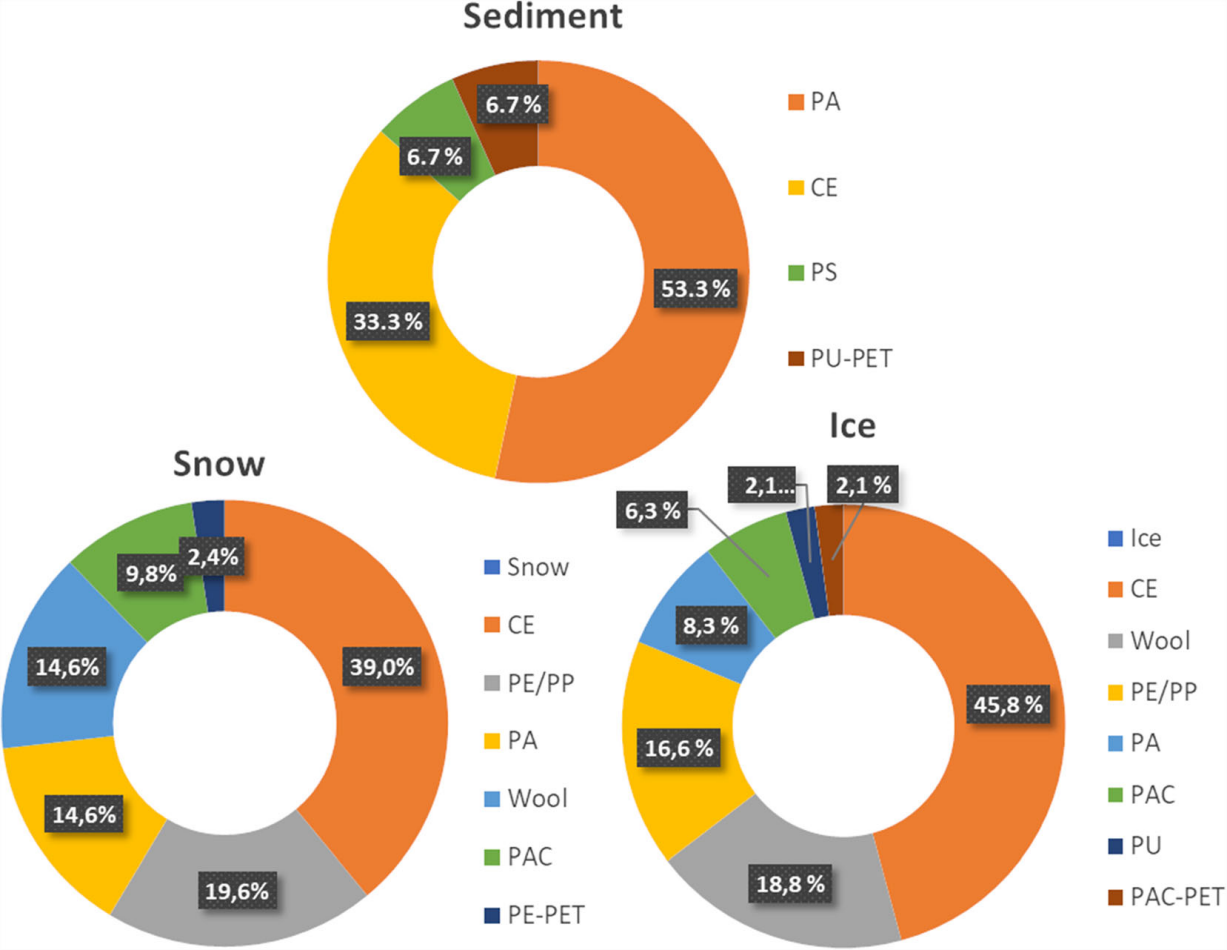
Percentage distribution of MPs. Percentage distribution of MP polymeric composition found in sediment, snow, and ice samples from lake Vesijärvi and Pikku Vesijärvi pond. Data represent the average percentages of MPs

In the sediments, the relative abundance of each type of analysed item was as follows: polyamides (PA) (53.3%), cellulose (CE) (most likely cotton and rayon) (33.3%), polystyrene (PS) (6.7%), and a blend polyurethane-polyethylene terephthalate (PU-PET) (6.7%) (Fig. 1).

In snow samples, the most abundant material detected was cellulose (39.0%) followed by polyethylene (PE) and polypropylene (PP) (19.6%), polyamides (14.6%), wool (14.6%), polyacrylates (PAC) (9.8%), and a blend polyethylene-polyethylene terephthalate (PE-PET) (2.4%) (Fig. 1).

Similar to the snow samples, cellulose was found to be the most prevalent material also in the ice matrix with a relative abundance of 45.8%, followed by wool (18.8%), polyethylene (PE) and polypropylene (16.6%), polyamides (8.3%), polyacrylates (6.3%), polyurethane (2.1%), and a blend polyacrylate-polyethylene terephthalate (PAC-PET) (2.1%) (Fig. 1).

All the items categorised as wool and cellulose were not included in the MP category, even though the cellulose category was most likely constituted by cotton and rayon, and the latter is a semisynthetic material used in clothing and cigarette filters (Barnes et al. 2009; Lusher et al. 2013).

Non-aged cellulose red fibres were excluded from the statistics as their state indicated that they had not been in the environment for a long time, and they most likely originated from the red coveralls.

Figures 2, 3, 4, 5, 6, 7, 8, and 9 show the representative study cases. When needed for the discussion, besides the FTIR spectrum of a fragment or fibre, the background of the filter substrate is also reported, representative of pixels (5.5 × 5.5 μm^2^ each) neighbouring the fibre or fragment in the map.

**Fig. 2:**
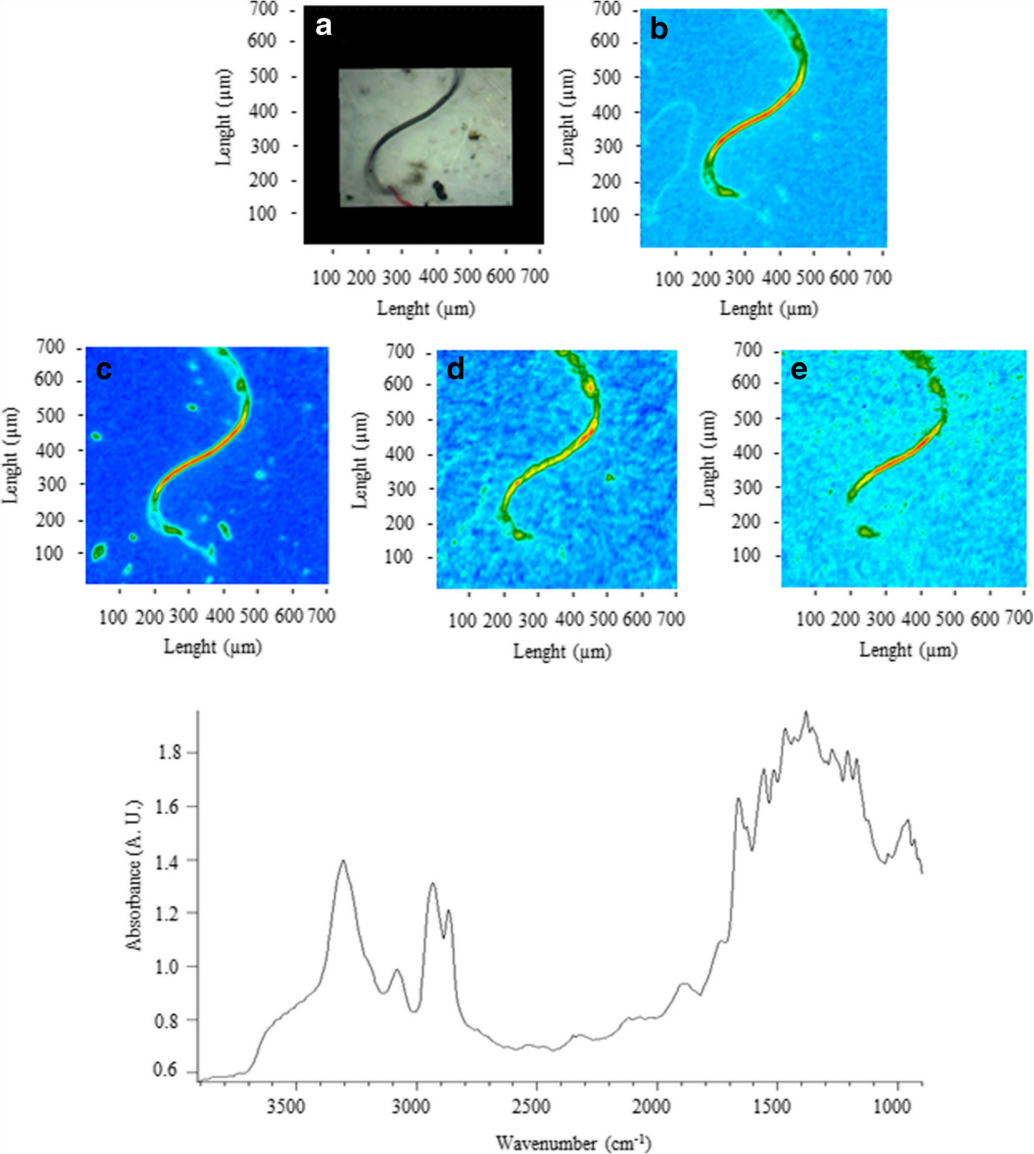
FTIR spectra, visible, and IR imaging of a polyamide fibre. (Top panel) visible light map of the filter substrate, with a plastic fibre lying on it (**a**). (Top right and bottom panels) 2D FTIR imaging maps, where the intensity of the following bands was mapped: 3403–3225 (NH stretching region) (**b**), 2908 (CH stretching) (**c**), 1724–1596 (C=O stretching) (**d**), and 1598–1528 (N–H bending, C–N stretching) cm^−1^ (**e**). The chromatic scale of each map qualitatively shows the absorbance intensity as follows: blue, green, yellow, and red. Maps have dimensions of 700 × 700 μm^2^. The bottom panel shows the FTIR reflectance spectrum of the plastic fibre, which relates to a single pixel (5.5 × 5.5 μm^2^) of the 2D imaging maps

**Fig. 3:**
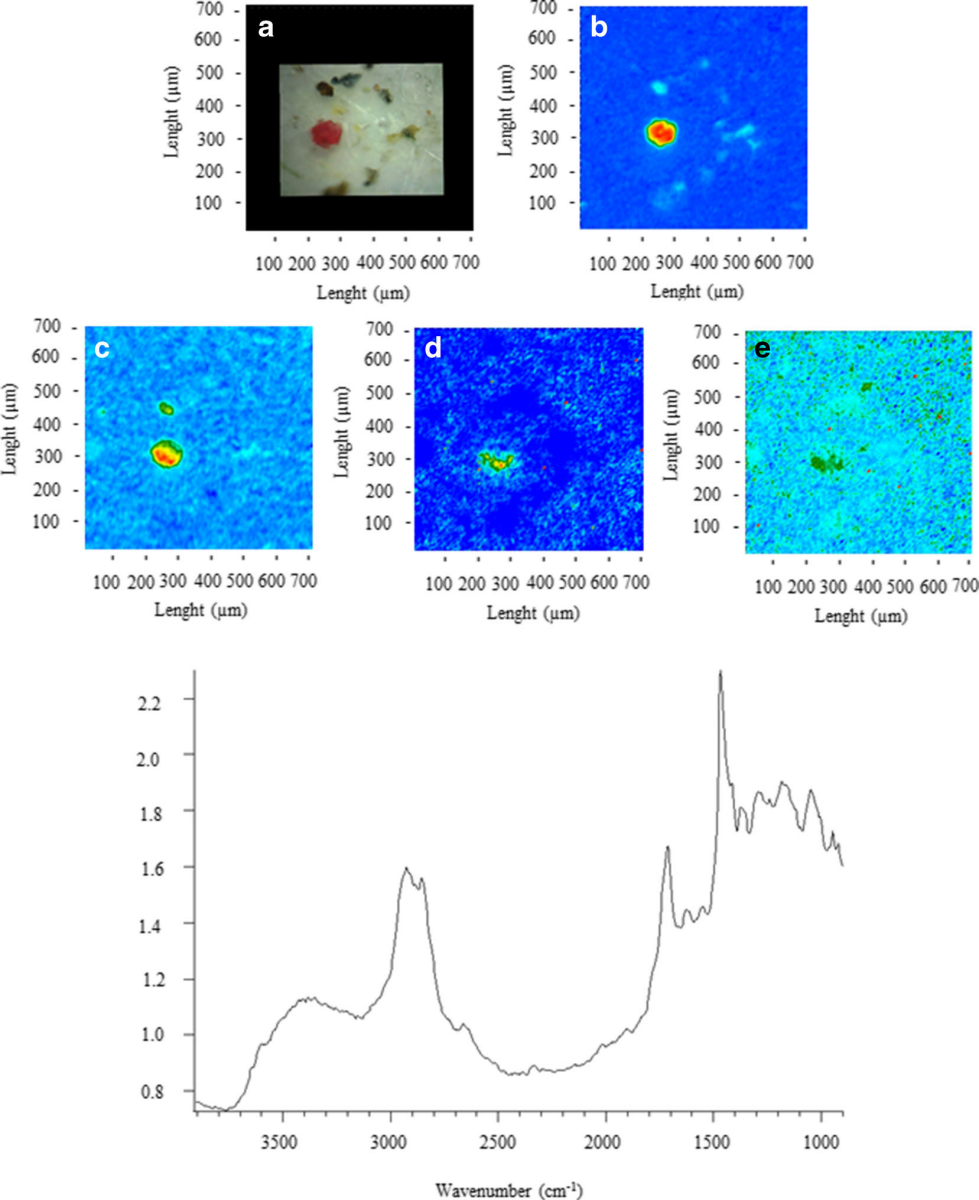
FTIR spectra, visible, and IR imaging of a polyethylene fragment. (Top left) Visible light map of the filter substrate, with a plastic fragment lying on it (**a**). (Top right and centre panels) 2D FTIR imaging maps, where the intensity of the following bands was mapped: 2923 (CH stretching) (**b**), 1708 (C=O stretching) (**c**), 1468 (δ_as_ CH_2_), and 1367 (δ_s_ CH_3_) cm^−1^. The chromatic scale of each map qualitatively shows the absorbance intensity as follows: blue, green, yellow, and red. Maps have dimensions of 700 × 700 μm^2^. The bottom panel shows the FTIR reflectance spectra of the plastic microfibre, which relates to a single pixel (5.5 × 5.5 μm^2^) of the 2D imaging maps

**Fig. 4:**
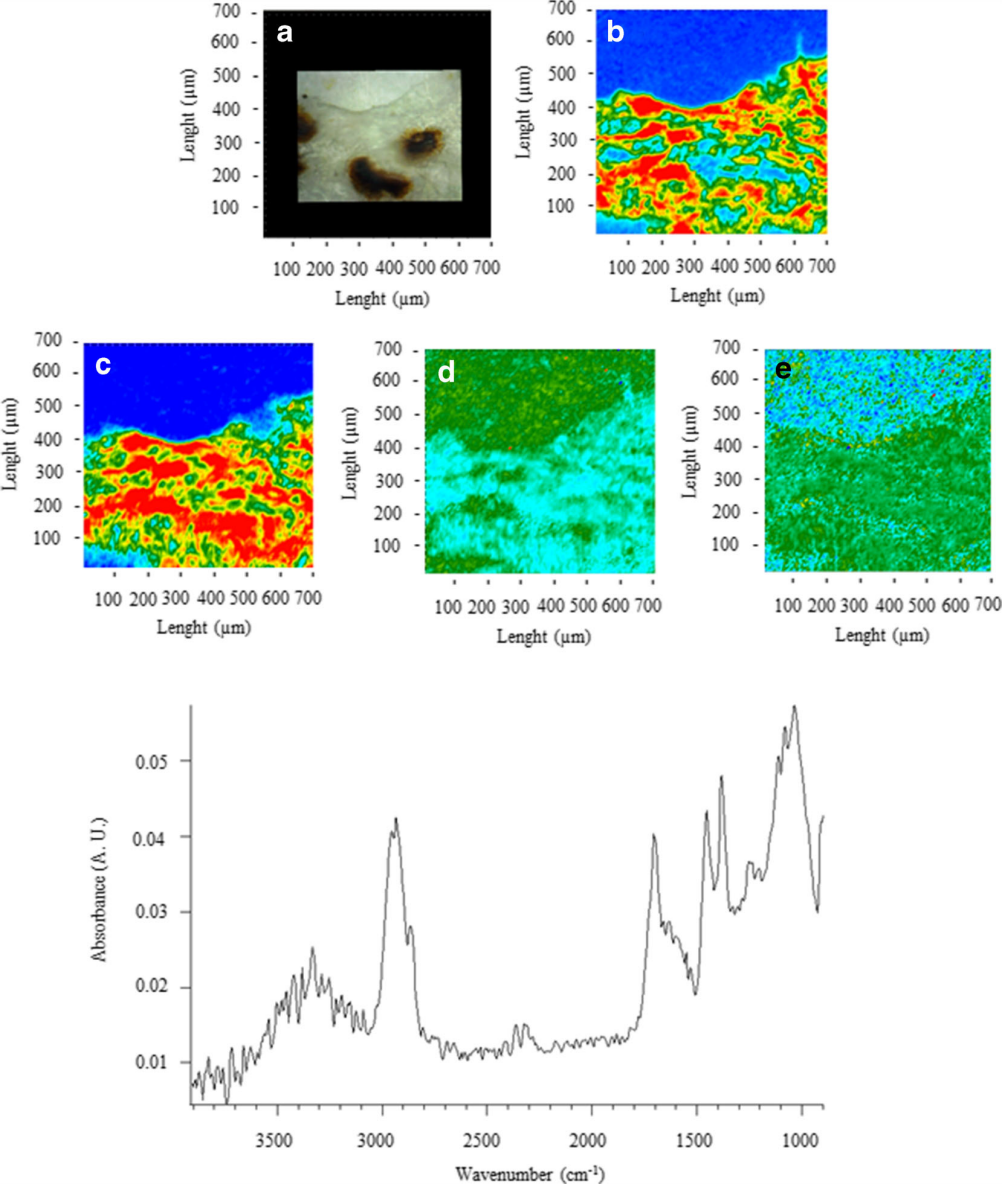
FTIR spectra, visible, and IR imaging of a polypropylene fragment. (Top left) Visible light map of the filter substrate, with a plastic fragment lying on it (**a**). (Top right and centre panels) 2D FTIR imaging maps, where the intensity of the following bands was mapped: 2931 (CH stretching) (**b**), 1715 (C=O stretching) (**c**), 1458 (δ_as_ CH_2_), and 1377 (δ_s_ CH_3_) cm^−1^. The chromatic scale of each map qualitatively shows the absorbance intensity as follows: blue, green, yellow, and red. Maps have dimensions of 700 × 700 μm^2^ (1 tick = 50 μm). The bottom panel shows the FTIR reflectance spectra of the plastic microfibre and the filter substrate. Each spectrum relates to a single pixel (5.5 × 5.5 μm^2^) of the 2D imaging maps

**Fig. 5:**
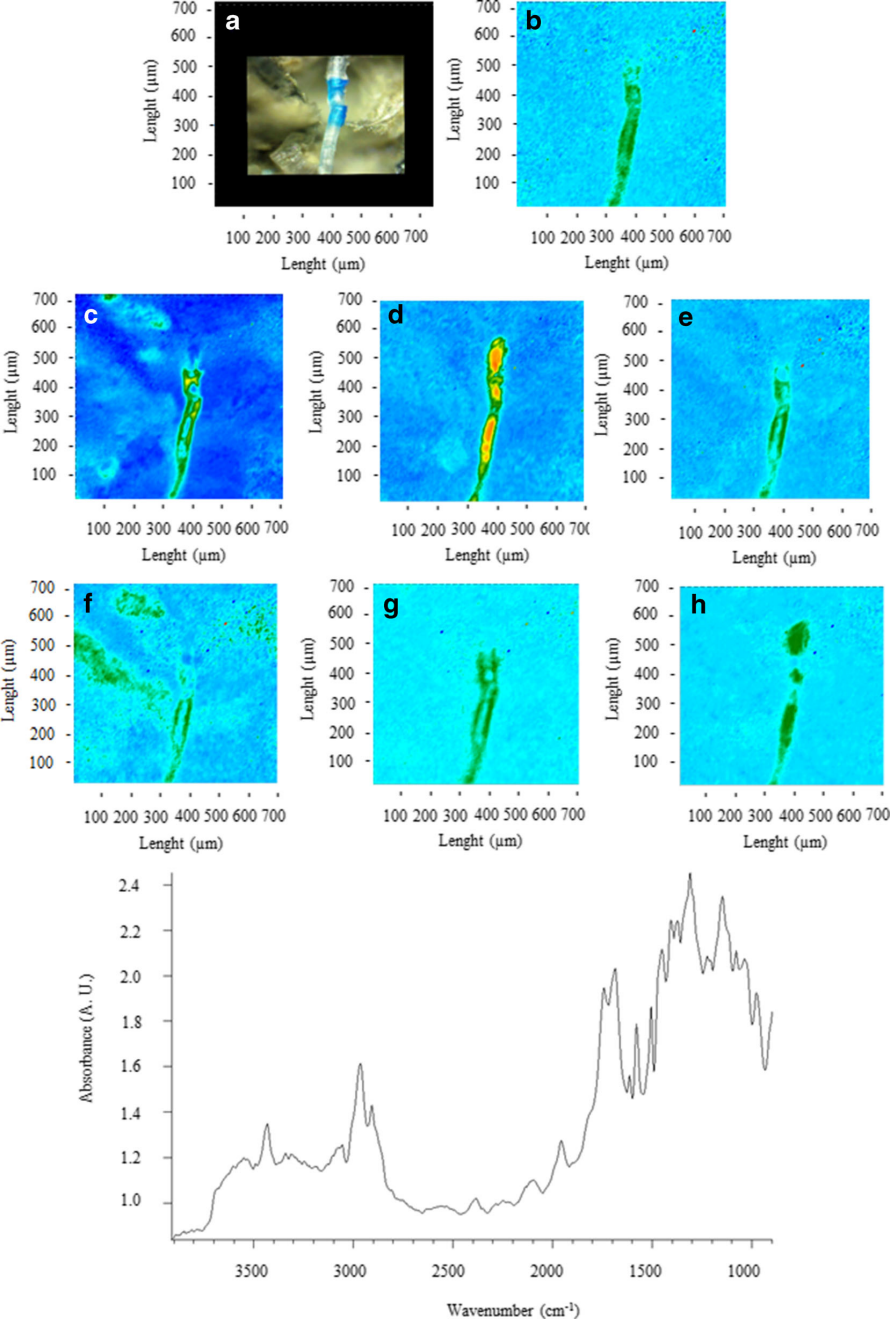
FTIR spectra, visible, and IR imaging of a polyurethane fibre. (Top left) Visible light map of the filter substrate, with a plastic fibre lying on it (**a**). (Top right, left, and centre panels) 2D FTIR imaging maps, where the intensity of the following bands was mapped: 3062 (aromatic CH stretching region) (**b**), 2962 (CH stretching) (**c**), 1743 (C=O stretching) (**d**), 1573 (C–N stretching), (**e**), 1504 (amide II) (**f**), 1446 (CH_2_ bending) (**g**), and 1303 (HCC stretching) cm^−1^ (**h**). The chromatic scale of each map qualitatively shows the absorbance intensity as follows: blue, green, yellow, and red. Maps have dimensions of 700 × 700 μm^2^. The bottom panel shows an FTIR reflectance spectrum of the plastic fragment, which relates to a single pixel (5.5 × 5.5 μm^2^) of the 2D imaging maps

**Fig. 6:**
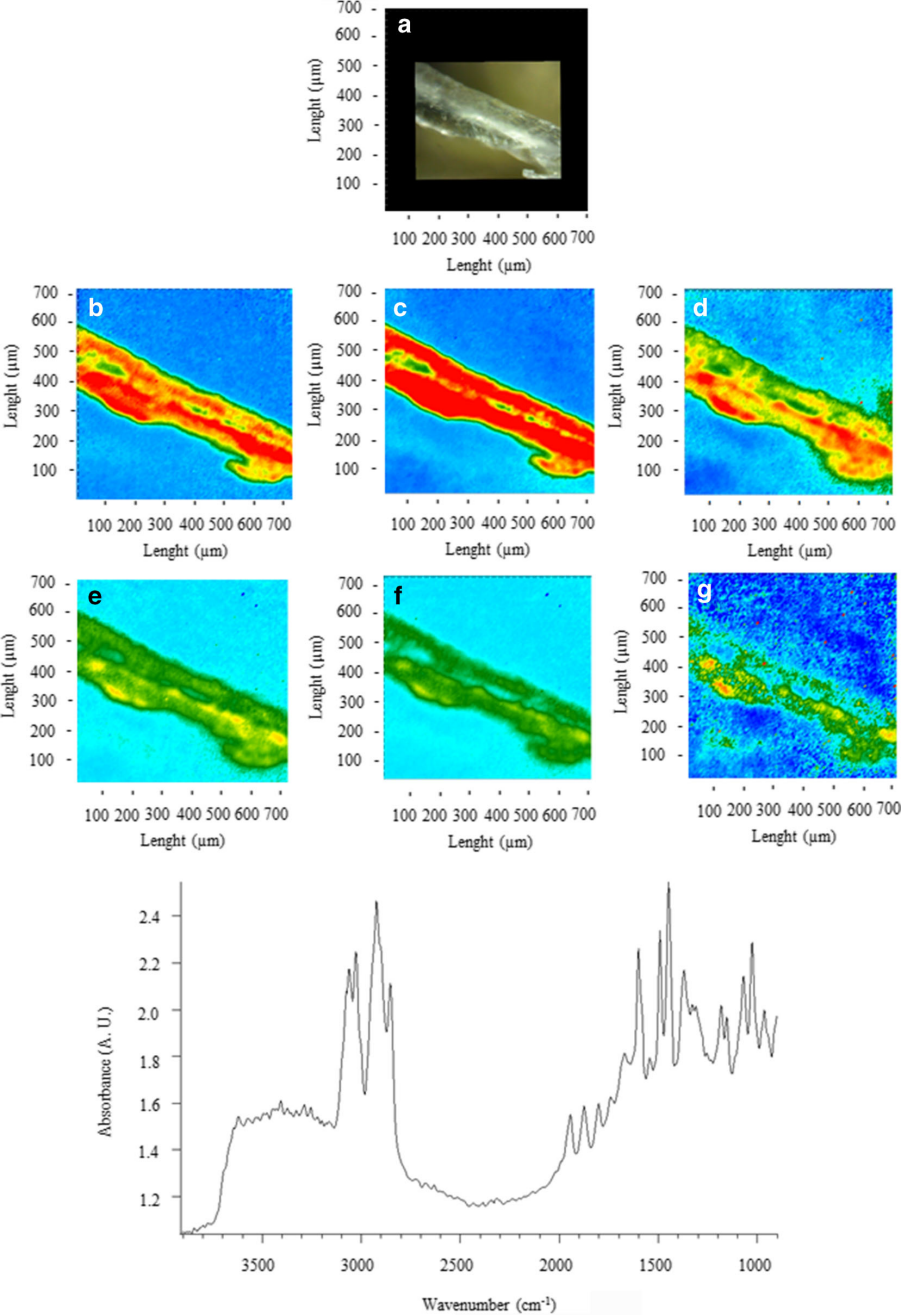
FTIR spectra, visible, and IR imaging of a polystyrene fibre. (Top panel) visible light map of the filter substrate, with a plastic fibre lying on it (**a**). (Top right and bottom panels) 2D FTIR imaging maps, where the intensity of the following bands was mapped: 3024 (aromatic C–H stretching vibrations) (**b**), 2908 (CH_2_ stretching) (**c**), 1600 (in-plane ring breathing) (**d**), 1492 and 1446 (carbon–carbon stretching vibrations in the aromatic ring) (**e**, **f**), and 1018 (aromatic CH bending) cm^−1^ (**g**). The chromatic scale of each map qualitatively shows the absorbance intensity as follows: blue, green, yellow, and red. Maps have dimensions of 700 × 700 μm^2^. The bottom panel shows the FTIR reflectance spectrum of the plastic fibre, which relates to a single pixel (5.5 × 5.5 μm^2^) of the 2D imaging maps

**Fig. 7:**
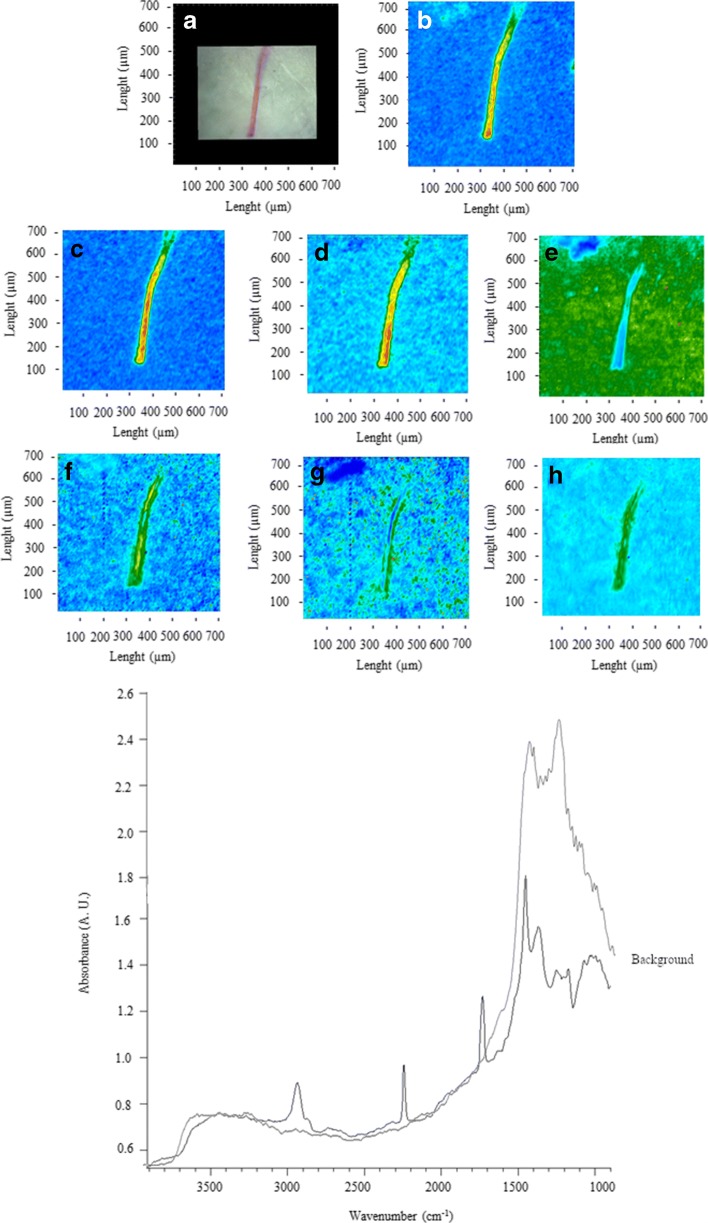
FTIR spectra, visible, and IR imaging of a polyacrylonitrile fibre. (Top panel) Visible light map of the filter substrate, with a plastic fibre lying on it (**a**). (Top right and bottom panels) 2D FTIR imaging maps, where the intensity of the following bands was mapped: 2920 (CH stretching) (**b**), 2240 (C–N stretching) (**c**), 1735 (CO stretching) (**d**), 1450 and 1368 (CH_2_ bending) (**e**, **f**), 1234 and 1071 cm^−1^ (C–O stretching) (**g**, **h**). The chromatic scale of each map qualitatively shows the absorbance intensity as follows: blue, green, yellow, and red. Maps have dimensions of 700 × 700 μm^2^. The bottom panel shows the FTIR reflectance spectra of the plastic fibre and the neighbouring filter. Each spectrum relates to a single pixel (5.5 × 5.5 μm^2^) of the 2D imaging maps

**Fig. 8:**
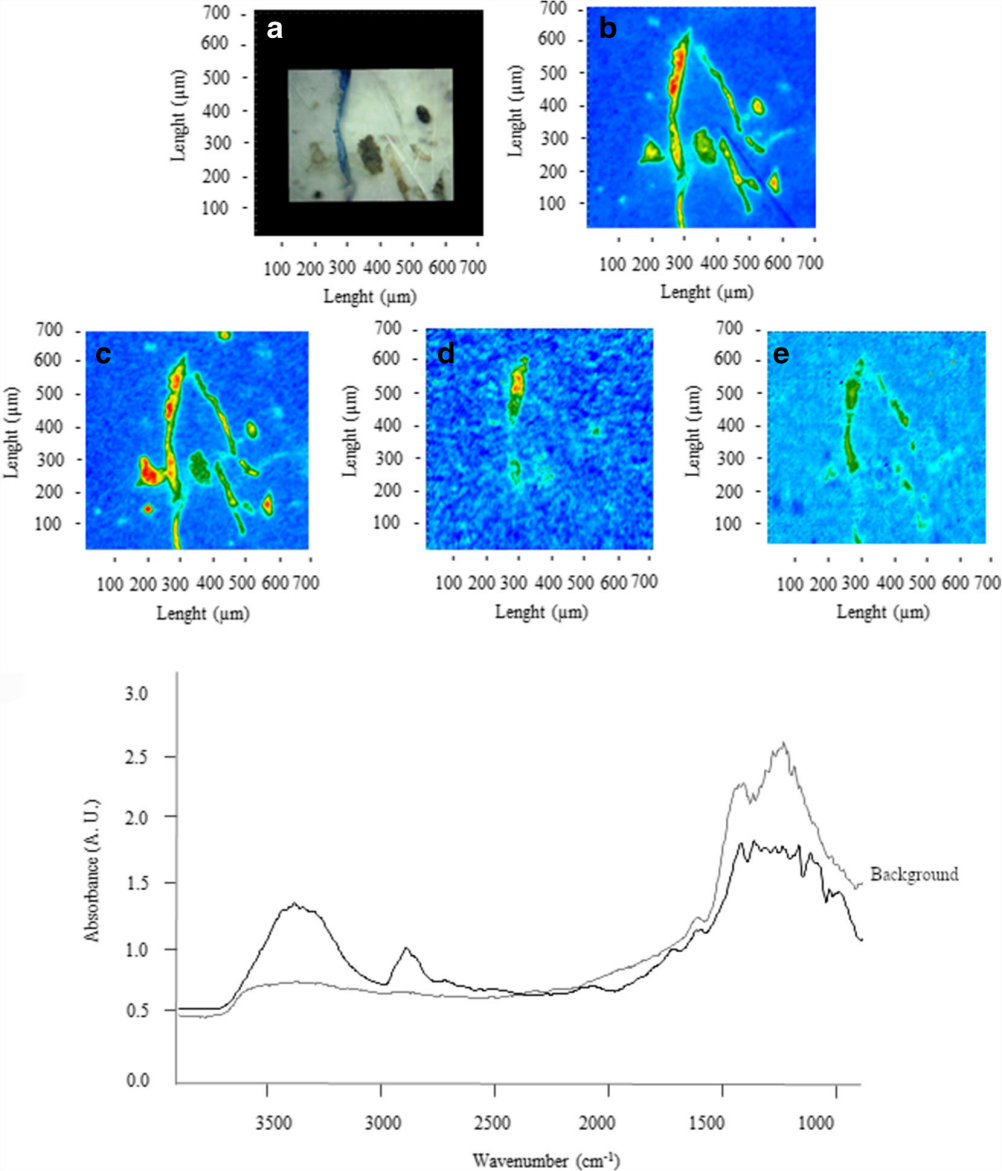
FTIR spectra, visible, and IR imaging of a cellulose fibre. (Top panel) Visible light map of the filter substrate, with a plastic fibre lying on it (**a**). (Top right and bottom panels) 2D FTIR imaging maps, where the intensity of the following bands was mapped: 3363 (OH stretching) (**b**), 2904 (CH stretching) (**c**), 1620 (OH bending) (**d**), and 1130 and 1078 cm^−1^ (C–O stretching) (**e**). The chromatic scale of each map qualitatively shows the absorbance intensity as follows: blue, gr.een, yellow, and red. Maps have dimensions of 700 × 700 μm^2^. The bottom panel shows the FTIR reflectance spectrum of the plastic fibre and the neighbouring filter. Each spectrum relates to a single pixel (5.5 × 5.5 μm^2^) of the 2D imaging maps

**Fig. 9:**
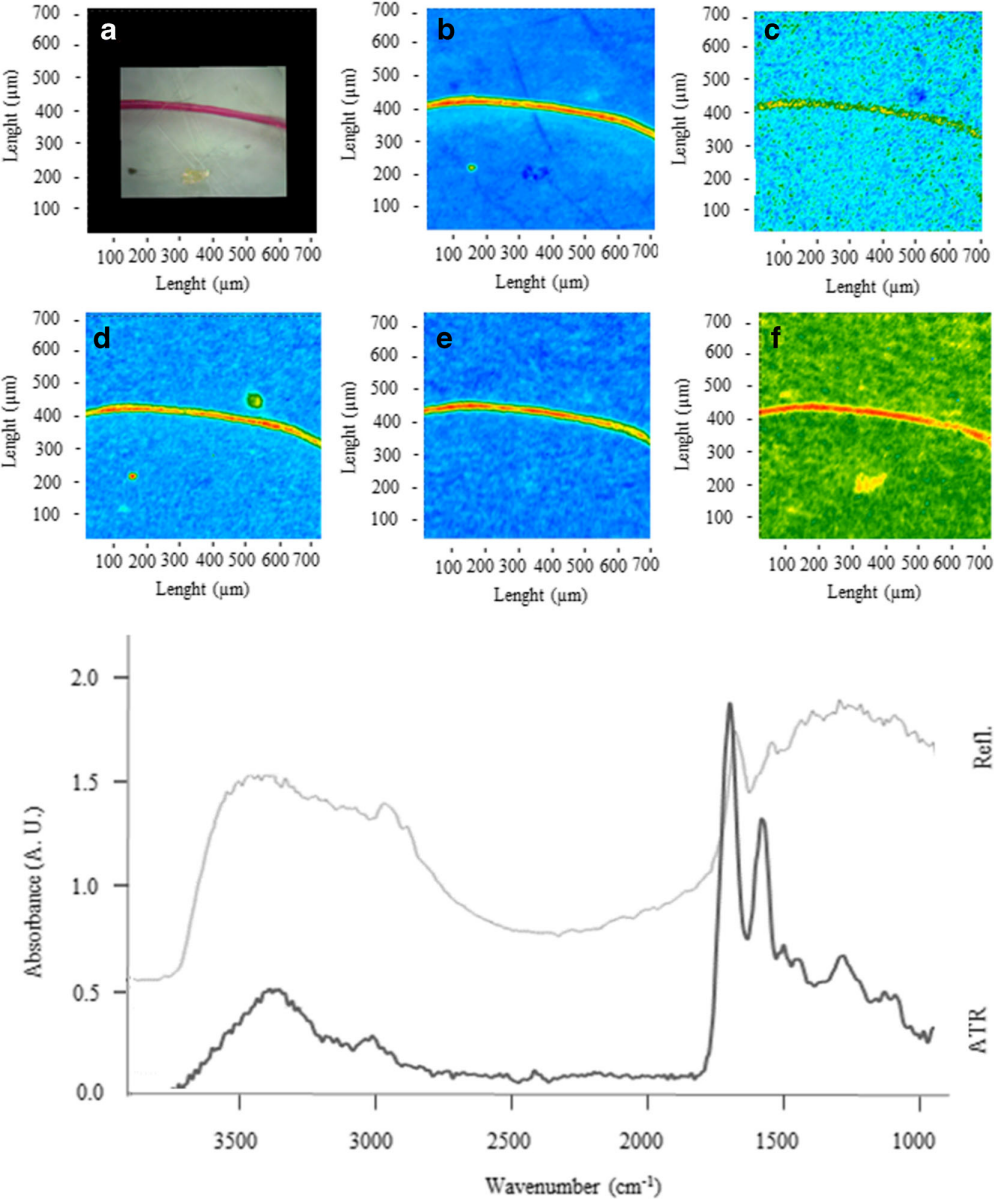
FTIR spectra, visible, and IR imaging of a wool fibre. (Top left) Visible light map of the filter substrate, with a plastic fibre lying on it (**a**). (Top right, centre, and bottom panels) 2D FTIR imaging maps, where the intensity of the following bands was mapped: 3460 (OH stretching) (**b**), 3047 (amide B stretching) (**c**), 2912 (CH stretching) (**d**), 1697 (**e**) (amide I stretching), and 1558 cm^−1^ (amide II stretching) (**f**). The chromatic scale of each map qualitatively shows the absorbance intensity as follows: blue, green, yellow, and red. Maps have dimensions of 700 × 700 μm^2^. The bottom panel shows the FTIR reflectance and ATR spectra of the fibre, and the reflectance spectrum of the filter. Each spectrum relates to a single pixel (5.5 × 5.5 μm^2^ in reflectance mode, 1.1 × 1.1 μm^2^ in ATR mode) of the 2D imaging maps

The bands observable in the spectra of the filter substrate include absorptions at 3400–3100 (OH stretching cellulose, algae (Stehfest et al. 2005)), 3000–2800 (CH stretching, algae; calcium carbonate absorptions, sediment (Ricci et al. 2006)), 1660–1640 (OH bending, water in cellulose; amide I, proteins in algae), 1560–1300 (amide II, proteins; CH_2_ and CH_3_ bending of organic sediment; antisymmetric CO_3_^2−^ stretching, ν_3_ calcium carbonate), and 1200–1030 (Si–O–Si stretching, silicates; ν(C–O–C) of saccharides in algae) (Chen et al. 2014).

Figure 2 illustrates a polyamide fibre. The spectra show characteristic absorptions, clearly distinguishable from the filter’s bands, at 3298 (N–H stretching), 2908 (C–H stretching), 2858 (CH_2_ stretching), 1634 (amide I stretching), and 1538 (C(O)–N–H bending, C–N stretching) cm^−1^ (Mahdi 2011; Pockett 2004; Porubuska et al. 2012).

The intensities of the characteristic peaks were imaged, showing IR maps in good agreement with the fibre profile in the visible image. No carbonyl absorptions were detected, excluding the presence of phthalates as additives.

Overall, 53.3%, 14.6%, and 8.3% of the investigated MPs were identified as polyamides in sediment, snow, and ice samples, respectively. The occurrence of these type of polymers in the two lakes can originate from the release of fibres stemming out from synthetic clothes of people doing recreational and sports activities on the frozen surfaces and from the discharge of the stormwater from the centre of the city of Lahti. Napper and Thompson (2016) have shown that the input of wastewaters from sewage treatment plants can represent a source of MPs, pointing out that a 6-kg wash load of acrylic clothes could release more than 700,000 fibres.

Figure 3 shows a fragment identified as PE. The characteristic bands assigned to PE are the following: 2915 (ν_as_ CH_2_), 2845 (ν_s_ CH_2_), 1468 (bending deformation), and 1367 (wagging deformation) cm^−1^ (Gulmine et al. 2002). The band at 1715 (C=O stretching ketones, carboxylic acids) suggests the presence of oxygenated groups (Gardette et al. 2013), likely due to the abiotic oxidation of polymer (Gewert et al. 2015). The spectra show absorption also at 2923 and 2864 (CH_2_ stretching), 1234 (ester group stretching), and 1068 (CH_2_ deformation) cm^−1^ which are characteristic of polyester (e.g. poly (ethylene terephthalate) (Chen et al. 2012)). These absorptions, together with the band at 1715 cm^−1^ could indicate the presence of a PE-PET blend. However, the fragment was identified as oxidised PE since the intensities of these bands are relatively weak, and the bands were not homogeneously detected over the surface of the fragment.

Figure 4 shows an example of a fragment identified as polypropylene, due to intense absorption peaks between 3000 and 2800 cm^−1^ (CH stretching region), at 2845 (ν CH), 1458 (δ CH_2_), and 1377 (δ CH_3_) cm^−1^ (Andreassen 1999; Asensio et al. 2009; Verleye et al. 2001; Noda et al. 2007; Jung et al. 2018).

The band at 1715 (C=O stretching ketones, carboxylic acids) suggests the presence of oxygenated groups (Gardette et al. 2013), likely due to the abiotic oxidation of polymer (Gewert et al. 2015).

PE and PP polymers accounted for 19.6% and 16.7% in snow and ice samples, respectively. No PE or PP MPs were found in the sediments, probably because the polymer densities are lower than freshwater, and they tend to float on the surface rather than to sink (Lambert and Wagner 2018).

Fig. 5 shows a bicolour (blue and transparent) polyurethane fibre ca. 50 μm thick. The characteristic bands assigned to PU are the following: at 3062 (aromatic CH stretching region, 2962 (C–H stretching), 1743 (C=O stretching), 1573 (C–N stretching), 1504 (amide II), 1446 cm^−1^ (CH_2_ bending), and 1303 (HCC stretching) cm (Bretzlaff and Sandlin 1987; Asegnejad et al. 2011; Wang et al. 2013; Jung et al. 2018). However, the presence of the bands at 1955 (combination band), 1713 (C=O stretching), and 1241 (C–O stretching) cm^−1^ suggests a blend PU-PET (Liang and Krimm 1959; Djebara et al. 2012; Jung et al. 2018), and this is conceivable since PET conversion products act as monomers in the synthesis of polyurethanes (Jankauskaite et al. 2008).

The sixth type of MPs found on the filter is shown in Fig. 6. The spectra collected on the fibre show absorptions at 3024 (aromatic C–H stretching vibrations), 2908 and 2847 (CH_2_ stretching), 1949 and 1878 (combination bands), 1600 and 1492 (aromatic ring stretching), and 1446 (CH_2_ bending) and 1018 (aromatic CH bending) cm^−1^ which are characteristic of polystyrene (Verleye et al. 2001; Noda et al. 2007; Asensio et al. 2009; Biazar et al. 2010; Olmos et al. 2014; Jung et al. 2018).

Figure 7 shows a fibre of ca. 900 μm length and about 40 μm thickness. The spectra collected on the fibre show intense absorptions at 2920 (CH stretching), 2240 (C–N stretching), and 1450 and 1368 (CH_2_ bending) cm^−1^ which are characteristics of polyacrylonitrile (Coates 2000; Verleye et al. 2001), as well as bands at 1735 (CO stretching) and 1234 and 1071 (C–O stretching) cm^−1^, which corresponds to the absorption of acrylates (Balamurugan et al. 2004; Duan et al. 2008; Ramesh et al. 2007; Jung et al. 2018). The seven peak intensities reported above were imaged, giving an IR map corresponding to the fibre profile in the visible light map. It must be noticed that, in the 2D imaging FTIR map corresponding to the letter D, the band at 1450 cm^−1^ is mapped as blue (low-intensity) pixels as compared to the pixels of the filter (green) surrounding the fibre. This can be explained observing the background filter, which shows that the filter presents high absorbance around 1450 cm^−1^.

Figure 8 shows a fibre of ca. 1100 μm length and about 30 μm thickness. The FTIR spectra present intense absorption at 3363 (OH stretching), 2904 (CH stretching), 1620 (OH bending), and 1130 and 1078 (C–O stretching) cm^−1^, characteristic of cellulose (i.e. cotton and rayon) (Garside and Wyeth 2003; Gaspar et al. 2014; Salama and El-Sakhawy 2016).

Figure 9 shows a non-synthetic microfibre of ca. 4000 μm length and about 35 μm thickness. The reflectance spectra exhibit intense protein bands at 3460 (OH stretching), 3047 (amide B), 1697 (amide I connected to C=O stretching), and 1558 (amide II deformating N–H and C–N stretching) cm^−1^ (Wojciechowska et al. 1999; Salama and El-Sakhawy 2016). However, the ATR spectra (acquired with a spatial resolution of 1.1 μm) proved fundamental to assign the fibre clearly, showing intense absorption at 3298 (amide A), 1639 (amide I connected to C=O stretching), 1519 (amide II C-N stretching and N-H bending), and 1226 (amide III C–N and C–O stretching, N–H, and O=C–N bending) cm^−1^ which are characteristic of wool (Wojciechowska et al. 1999; Salama and El-Sakhawy 2016). Wool fibres accounted for 14.6% and 18.8% in snow and ice samples, respectively. No wool fibres were detected in sediments.

### Microplastic occurrence and distribution

**Fig. 10:**
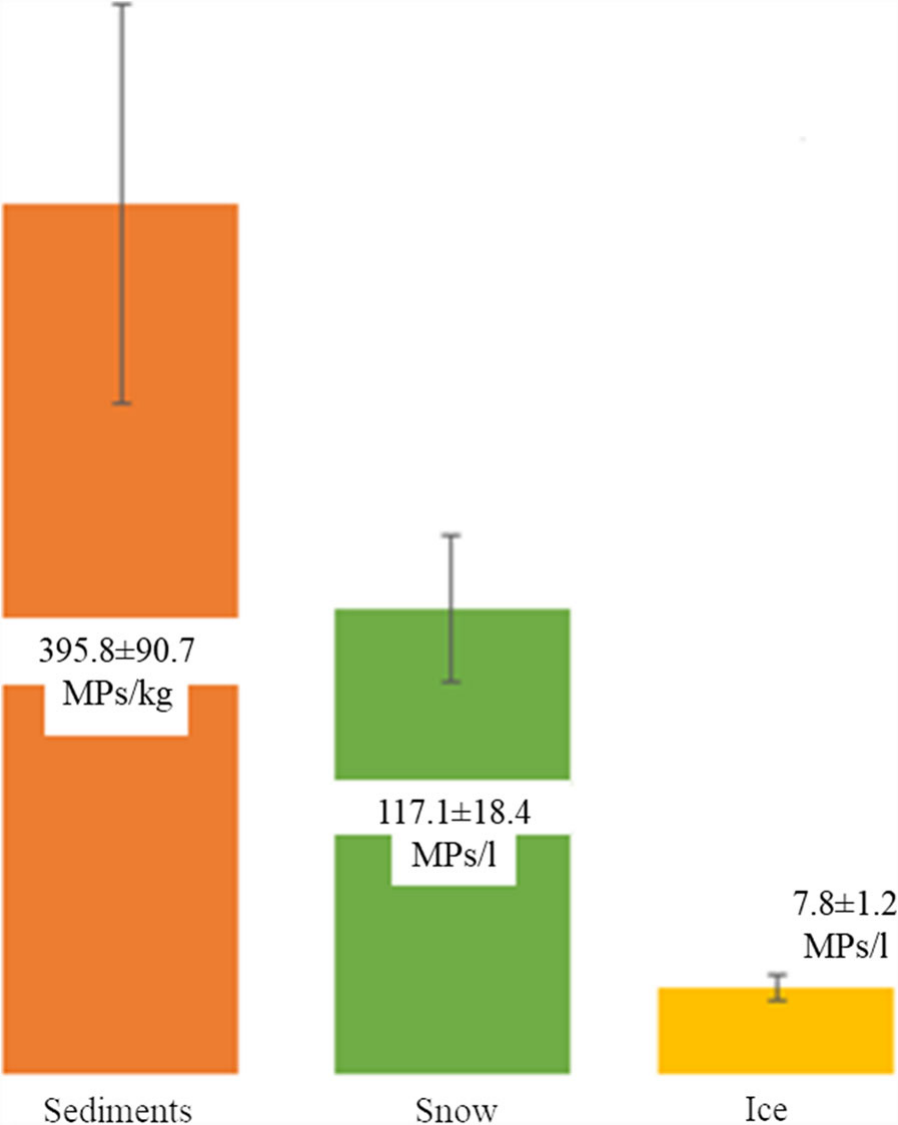
MP average concentrations found in sediment, snow, and ice

MPs were detected in all three sample matrices, and they were categorised into two principal classes, including fibres (average percentage 99.6 ± 0.5% in snow, 99.1 ± 0.7% in ice, 99.2 ± 1.9% in sediments) and fragments (average percentage 0.4 ± 0.5% in snow, 0.9 ± 0.7% in ice, 0.8 ± 1.9% in sediments). No particles categorised as “film”, “granules”, or “pellets” were found (Zhao et al. 2015). MP mean concentrations found in sediment, snow, and ice were 395.8 ± 90.7 MPs/kg, 117.1 ± 18.4 MPs/L), and 7.8 ± 1.2 MPs/L, respectively (Fig. 10). Besides, synthetic items, cellulose, and wool fibres were also found in the three different matrices.

Our results on MP concentration in sediments (average 395.8 ± 90.7 MPs/kg) were in compliance with what is reported in the scientific literature in Venice Lagoon sediments and Rhine river sediments ranging from 672 to 2175 MPs/kg and from 228 to 3763 MPs/kg, respectively (Vianello et al. 2013; Klein et al. 2015; Lusher 2015).

The abundance of MPs in ice samples are significantly lower than those detected in the Arctic Sea ice by Bergmann et al. (2017), which reported mean concentrations of 2 × 10^3^ particles/L in pack ice and 6 × 10^2^ particles/L, respectively, but higher than those reported by Obbard et al. (2014) for Arctic Sea ice, which ranged from 0.038 to 0.234 particles/L. The MP detected in ice in the present study is also comparable to the mean abundance of MPs found in other urban aquatic systems like in the Los Angeles River and in lake Taihu surface water where the reported mean was 12.932 pieces/L and a range between 3 and 26 MPs/L, respectively (Moore et al. 2011; Su et al. 2016).

Regarding snow samples, it was not possible to make any data comparison, as there are no published data on the occurrence of MPs in a snow matrix. MP concentration in snow is one order of magnitude higher than that detected in ice, suggesting that the high porosity of the snow matrix could play an important role in trapping MP airborne fibres.

## Conclusions

Our results showed the presence of synthetic fibres (≥ 99%) and fragments (≤ 1%) in all three sample matrices, which highlight the potential impact arising from the release of fibres stemming out from clothes of people doing recreational and sports activities on the lakes and from the stormwater collected from the city centre of Lahti. These factors could represent a remarkable source of MPs that end up in freshwater environment posing a risk for the biota and likely entering the food web.

## Data Availability

The data that support the findings of this study are available from the corresponding author upon reasonable request.
